# Urinary Sodium and Potassium, and Risk of Ischemic and Hemorrhagic Stroke (INTERSTROKE): A Case–Control Study

**DOI:** 10.1093/ajh/hpaa176

**Published:** 2020-11-16

**Authors:** Conor Judge, Martin J O’Donnell, Graeme J Hankey, Sumathy Rangarajan, Siu Lim Chin, Purnima Rao-Melacini, John Ferguson, Andrew Smyth, Denis Xavier, Liu Lisheng, Hongye Zhang, Patricio Lopez-Jaramillo, Albertino Damasceno, Peter Langhorne, Annika Rosengren, Antonio L Dans, Ahmed Elsayed, Alvaro Avezum, Charles Mondo, Danuta Ryglewicz, Anna Czlonkowska, Nana Pogosova, Christian Weimar, Rafael Diaz, Khalid Yusoff, Afzalhussein Yusufali, Aytekin Oguz, Xingyu Wang, Fernando Lanas, Okechukwu S Ogah, Adesola Ogunniyi, Helle K Iversen, German Malaga, Zvonko Rumboldt, Shahram Oveisgharan, Fawaz Al Hussain, Salim Yusuf

**Affiliations:** Department of Medicine, NUI Galway, Galway, Ireland; Department of Medicine, Population Health Research Institute, McMaster University and Hamilton Health Sciences, Hamilton, Ontario, Canada; Wellcome Trust Health Research Board Irish Clinical Academic Training (ICAT), Dublin, Ireland; Department of Medicine, NUI Galway, Galway, Ireland; Department of Medicine, Population Health Research Institute, McMaster University and Hamilton Health Sciences, Hamilton, Ontario, Canada; School of Medicine and Pharmacology, Faculty of Health and Medical Sciences, University of Western Australia, Perth, Western Australia, Australia; Department of Medicine, Population Health Research Institute, McMaster University and Hamilton Health Sciences, Hamilton, Ontario, Canada; Department of Medicine, Population Health Research Institute, McMaster University and Hamilton Health Sciences, Hamilton, Ontario, Canada; Department of Medicine, Population Health Research Institute, McMaster University and Hamilton Health Sciences, Hamilton, Ontario, Canada; Department of Medicine, NUI Galway, Galway, Ireland; Department of Medicine, NUI Galway, Galway, Ireland; Department of Medicine, St John’s Medical College and Research Institute, Bangalore, India; Department of Medicine, National Center of Cardiovascular Disease, Beijing, China; Department of Medicine, Beijing Hypertension League Institute, Beijing, China; Department of Medicine, Instituto de Investigaciones MASIRA, Universidad de Santander, Bucaramanga, Colombia; Faculty of Medicine, Eduardo Mondlane University, Maputo, Mozambique; Department of Medicine, Glasgow Royal Infirmary, University of Glasgow, Glasgow, Scotland, UK; Department of Molecular and Clinical Medicine, University of Gothenburg and Region Västra Götaland, Sahlgrenska University Hospital, Gothenburg, Sweden; College of Medicine, University of Philippines, Manila, Philippines; Department of Surgery, Al Shaab Teaching Hospital, Khartoum, Sudan; Department of Medicine, International Research Center, Hospital Alemão Oswaldo Cruz, São Paulo, Brazil; Department of Medicine, Kiruddu National Referral Hospital, Kampala, Uganda; Military Institute of Aviation Medicine, Warsaw, Poland; Department of Medicine, Military Institute of Aviation Medicine, Warsaw, Poland; Department of Medicine, National Medical Research Center of Cardiology, Moscow, Russia; Department of Neurology, University Hospital, Essen, Germany; Department of Medicine, Estudios Clínicos Latino America (ECLA), Instituto Cardiovascular de Rosario (ICR), Rosario, Argentina; Department of Medicine, Universiti Teknologi MARA, Selayang, Selangor and UCSI University, Kuala Lumpur, Malaysia; Department of Medicine, Hatta Hospital, Dubai Health Authority/Dubai Medical College, Dubai, UAE; Department of Internal Medicine, Istanbul Medeniyet University, Istanbul, Turkey; Department of Medicine, Beijing Hypertension League Institute, Beijing, China; Faculty of Medicine, Universidad de La Frontera, Temuco, Chile; Department of Medicine, University College Hospital, Ibadan, Nigeria; Department of Medicine, University College Hospital, Ibadan, Oyo State, Nigeria; Department of Neurology, Rigshospitalet, University of Copenhagen, Denmark; School of Medicine, Universidad Peruana Cayetano Heredia, Lima, Peru; Department of Medicine, University of Split, Split, Croatia; Department of Medicine, Rush Alzheimer Disease Research Center in Chicago, Chicago, Illinois, USA; Department of Medicine, King Saud University, Riyadh, Saudi Arabia; Department of Medicine, Population Health Research Institute, McMaster University and Hamilton Health Sciences, Hamilton, Ontario, Canada

**Keywords:** blood pressure, hypertension, intracerebral hemorrhage, ischemic stroke, potassium, sodium, stroke

## Abstract

**BACKGROUND:**

Although low sodium intake (<2 g/day) and high potassium intake (>3.5 g/day) are proposed as public health interventions to reduce stroke risk, there is uncertainty about the benefit and feasibility of this combined recommendation on prevention of stroke.

**METHODS:**

We obtained random urine samples from 9,275 cases of acute first stroke and 9,726 matched controls from 27 countries and estimated the 24-hour sodium and potassium excretion, a surrogate for intake, using the Tanaka formula. Using multivariable conditional logistic regression, we determined the associations of estimated 24-hour urinary sodium and potassium excretion with stroke and its subtypes.

**RESULTS:**

Compared with an estimated urinary sodium excretion of 2.8–3.5 g/day (reference), higher (>4.26 g/day) (odds ratio [OR] 1.81; 95% confidence interval [CI], 1.65–2.00) and lower (<2.8 g/day) sodium excretion (OR 1.39; 95% CI, 1.26–1.53) were significantly associated with increased risk of stroke. The stroke risk associated with the highest quartile of sodium intake (sodium excretion >4.26 g/day) was significantly greater (*P* < 0.001) for intracerebral hemorrhage (ICH) (OR 2.38; 95% CI, 1.93–2.92) than for ischemic stroke (OR 1.67; 95% CI, 1.50–1.87). Urinary potassium was inversely and linearly associated with risk of stroke, and stronger for ischemic stroke than ICH (*P* = 0.026). In an analysis of combined sodium and potassium excretion, the combination of high potassium intake (>1.58 g/day) and moderate sodium intake (2.8–3.5 g/day) was associated with the lowest risk of stroke.

**CONCLUSIONS:**

The association of sodium intake and stroke is J-shaped, with high sodium intake a stronger risk factor for ICH than ischemic stroke. Our data suggest that moderate sodium intake—rather than low sodium intake—combined with high potassium intake may be associated with the lowest risk of stroke and expected to be a more feasible combined dietary target.

## Graphical Abstract

**Graphical Abstract:**
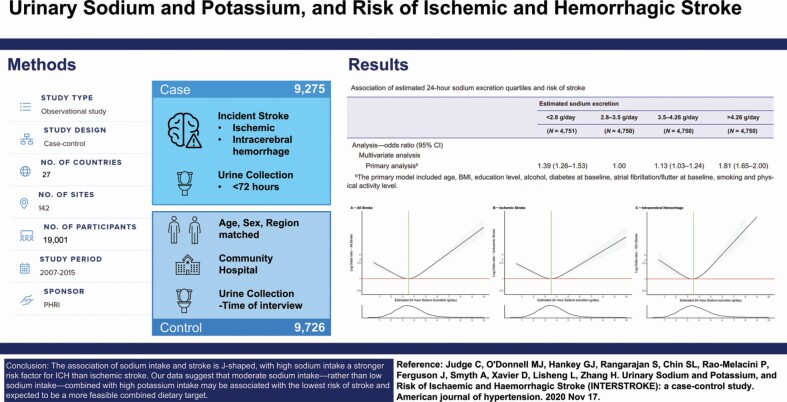

Hypertension is the key modifiable risk factor for stroke and increasing sodium intake is positively associated with blood pressure.^1,2^ Reduction of sodium intake, to low intake levels of 2 g/day or lower, has been proposed to be an effective population-level intervention to reduce blood pressure, and inferentially, reduce the burden of stroke.^3–5^ However, despite the modest positive association between sodium intake and blood pressure,^6^ the pattern of association of sodium intake with cardiovascular disease is consistently J-shaped in a number of large epidemiologic studies,^7–9^ despite using different methods to estimate sodium intake (24-hour urine collection, early morning fasting samples, or random nonfasting urine samples). For stroke, individual studies report an inconsistent relationship between sodium intake and stroke, with different epidemiologic studies reporting a linear association, a curvilinear relationship, or J-shaped association.^10^ In addition, the association of high sodium intake with stroke persists in most observational studies, after adjusting for blood pressure, suggesting mechanisms other than blood pressure may also mediate the increased cardiovascular risk.^7^

Considerable public health efforts and resources are being invested in targeting low sodium intake (<2 g/day),^11^ although there is controversy about whether low sodium intake represents the optimal target for cardiovascular prevention. Moreover, the feasibility of a combined target of low sodium and high potassium intake is challenged because only a very small proportion of the population consume this joint electrolyte target^12,13^; sodium and potassium intake usually correlate positively with each other indicating that targeting low sodium intake is more likely to be associated with reductions in potassium intake among free-living individuals, and vice versa.^14^ Increased potassium intake appears to be an important target for stroke prevention, with meta-analyses reporting a linear reduction in stroke risk associated with increased potassium intake.^15^ Studies also suggest that the adverse cardiovascular effects of high sodium intake may be mitigated with high potassium intake.^12,16,17^ Therefore, evaluating the association of sodium intake with stroke necessarily requires a combined analysis of the relationship of both electrolytes with stroke risk overall, and within stroke subtypes.^17^

INTERSTROKE was a standardized international case–control study that included participants from 30 countries.^1^ The unique aspects of this observational study are the breadth of the international population included, the standardized measurement of vascular risk factors (including diet) and the valid determination of primary stroke subtype (ischemic or intracerebral hemorrhage [ICH]) using neuroimaging.

In this paper, we report the individual, and joint, associations of estimated sodium and potassium excretion (surrogates for intake) with stroke and its subtypes.

## METHODS

### Study design and participants

INTERSTROKE is a large, international case–control study of risk factors for first stroke. 13,462 stroke patients and 13,483 matched controls were recruited between 11 January 2007 and 8 August 2015. For the current analyses, we include 9,275 cases and 9,726 controls with urinary measures of sodium and potassium (8,761 matched pairs for conditional analysis). Each case was matched for sex and age (±5 years) with controls (Supplementary Table S1 online). Cases were patients with first acute stroke, either ischemic or ICH, with confirmation by computed tomography or magnetic resonance imaging brain imaging. Patients with stroke were enrolled within 5 days of symptom onset and within 72 hours of hospital admission. Stroke severity was measured using the modified Rankin scale at the time of recruitment and at 1-month follow-up. The study was approved by the ethics committees in all participating centers. Written informed consent was obtained from participants or their proxy.

### Measurement of risk factors

Standardized questionnaires were used to collect data on demographics, lifestyle stroke risk factors, and characteristics of acute stroke from all cases and controls (Supplementary Table S2 online). Physical measurements of weight, height, waist and hip circumferences, heart rate, and blood pressure were recorded in a standardized manner. In cases, blood pressure and heart rate were measured at 3 time-points: at admission, the next morning, and at the time of interview. A modified Rankin scale score was collected at 3 time-points for cases: preadmission, time of interview and at 1-month follow-up (either in person or by phone), and 1 time-point for controls (time of interview). Ischemic stroke subtype was based on clinical assessment (baseline and 1-month), neuroimaging (baseline), and results of tests to determine etiology (ultrasound of carotids, cardiac imaging, and cardiac monitoring). Hypertension was defined as a composite of self-reported hypertension and a blood pressure reading of greater than 140/90 mm Hg at recruitment. Diabetes mellitus was defined as self-reported diabetes or a HbA1c of greater than 6.5% at recruitment.

### Blood and urine collection and analysis

Nonfasting blood and urine samples were taken from cases within 72 hours of recruitment and controls (at the time of interview), frozen at −20° to −70° and shipped to core laboratories (Hamilton-Canada, Beijing-China, Bangalore-India, and Istanbul-Turkey). Several formulae exist for estimation of 24-hour sodium and potassium excretion from spot urinary sodium/potassium measurements.^18–20^ These formulae have been validated against 24-hour urine collections^21^ and serve as a valid measure of mean population sodium and potassium intake.^22^ The Tanaka formula was used to estimate 24-hour urine sodium and potassium excretion^18^ and is reported to be associated with the least biased estimate for casual (nonfasting) urine samples in an international population.^21^

### Statistical analysis

We calculated the correlation between urinary sodium and potassium excretion using Pearson’s correlation coefficient, and of sodium and potassium excretion with blood pressure using an intraclass correlation coefficient in controls (excluding those with known hypertension or taking diuretics). We used multivariable conditional logistic regression to evaluate the association of sodium and potassium excretion with stroke, employing restricted cubic-spline plots to explore the pattern of association.^23^ For analysis of categories of estimated sodium excretion, we set the reference group as the second quartile (2.8–3.5 g/day), as this was identified as the lowest risk category on initial univariate analyses, and consistent with the range of lowest risk on cubic splines. Similarly, we set the first quartile (<1.34 g/day) as the reference group for estimated potassium excretion.

We adjusted for covariates in 4 sequential models. Model 1 was adjusted for age and body mass index. Model 2 (the primary model) was additionally adjusted for education level (none-reference, 1–8 years, 9–12 years, Trade School, College/University), alcohol intake (never-reference, former, current), diabetes, atrial fibrillation or flutter, smoking (never-reference, former, current), and physical activity level (strenuous-reference, moderate, mild, mainly sedentary). Model 3 included all the variables in model 2 and added, estimated excretion of potassium (Tanaka) and the alternative healthy eating index dietary score as an overall measure of diet quality. Model 4 included hypertension status, mean systolic blood pressure, mean diastolic blood pressure, and medications which modify sodium excretion, which was a model to explore variables potentially along the causal pathway mediating the association of sodium and potassium intake with stroke. Model 4 was reproduced separately with the 3 components of the mean blood pressure variable: time of admission, the morning after admission, and during the interview. We examined the consistency of these associations by performing analyses in subgroups using our primary model (conditional analysis) based on key characteristics that might modify the association between sodium, potassium, and stroke (ethnicity, body mass index, sex, age, hypertension, and diuretic therapy), using the Wald test to assess statistical interactions. We excluded small subgroups (<500 participants).

We performed an analysis of the combined effects of sodium and potassium excretion, in which we generated 8 categories (4 × 2) by sodium excretion quartile (<2.8, 2.8–3.5, 3.5–4.26, and >4.26 g/day) and potassium excretion above and below median (1.58 g/day). We completed a sensitivity analysis in which we excluded patients (cases) with a modified Rankin score greater than 2, as such patients may more likely not consume their usual diets and may receive cointerventions (e.g., intravenous fluids and enteric feeding due to their disability). Given that time from hospital admission to sample collection may also affect the classification of intake categories, we completed an analysis that excluded participants with samples collected greater than 48 hours after admission. All analyses were performed using R version 3.5.3 (Great Truth).

### Role of the funding source

The study funders had no role in study design; in the collection, analysis, and interpretation of data; in the writing of the report; and in the decision to submit the paper for publication.

## RESULTS

### INTERSTROKE participants

**Figure 1.**
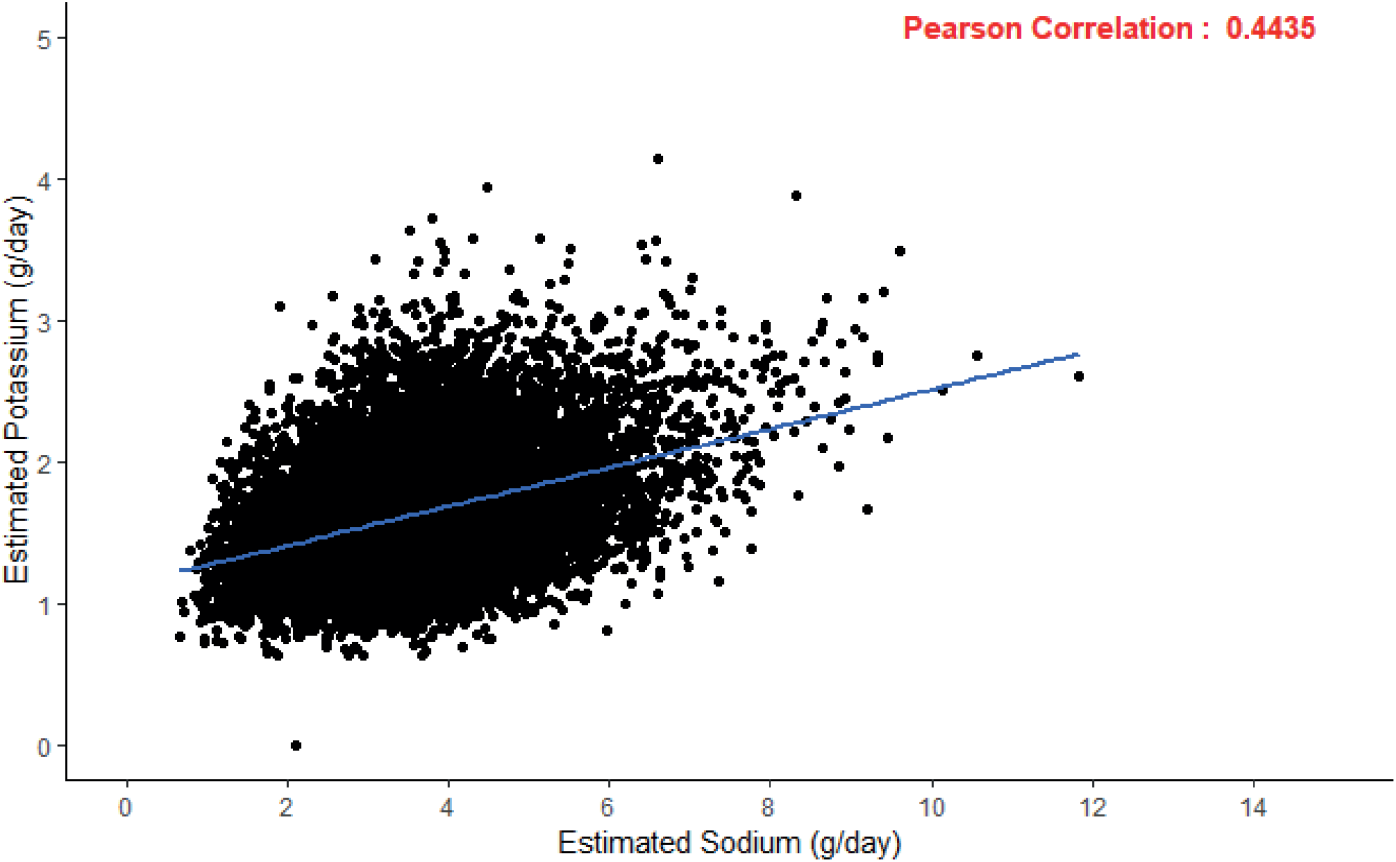
Scatterplot of estimated urinary sodium and potassium excretion.

Between 11 January 2007 and 8 August 2015, the INTERTSROKE study enrolled 9,275 cases of acute first stroke and 9,726 matched controls (8,761 matched pairs for conditional analysis) who also had urinary samples collected. Table 1 and Supplementary Table S3 online (sodium) and Supplementary Table S4 online (potassium) outline the characteristics of patients including comorbidities, stroke type, stroke severity, and blood pressure by quartiles of sodium and potassium excretion. The mean time between stroke onset and collection of urine sample was 2.08 ± 1.27 days and the mean time between hospitalization and collection of urine sample was 1.51 ± 1.04 days. Figure 1 reports the scatterplot and a statistically significant correlation (*R* = 0.4435, *P* < 0.0001) between estimated urinary sodium and potassium excretion for cases and controls in the INTERSTROKE population. The mean 24-hour sodium excretion per day were 3.69 g for cases and 3.54 g for control (*P* < 0.001) (Table 1). The mean 24-hour potassium excretion per day were 1.57 g for cases and 1.68 g for controls (*P* < 0.001) (Supplementary Table S4 online).

**Table 1.** Characteristics of the study participants at baseline, according to estimated sodium excretion (conditional analysis)

Characteristic	Case					Control				
	Estimated sodium excretion									
	All	<2.8 g/day	2.8–3.5 g/day	3.5–4.3 g/day	>4.3 g/day	All	<2.8 g/day	2.8–3.5 g/day	3.5–4.3 g/day	>4.3 g/day
	(*N* = 8,761)	(*N* = 2,214)	(*N* = 1,911)	(*N* = 2,065)	(*N* = 2,571)	(*N* = 8,767)	(*N* = 2,075)	(*N* = 2,481)	(*N* = 2,352)	(*N* = 1,859)
Estimated excretion, g/day										
Sodium	3.69 ± 1.28	2.23 ± 0.44	3.19 ± 0.20	3.88 ± 0.21	5.18 ± 1.02	3.54 ± 1.04	2.32 ± 0.41	3.19 ± 0.20	3.88 ± 0.21	4.93 ± 0.93
Potassium	1.58 ± 0.38	1.43 ± 0.30	1.49 ± 0.33	1.56 ± 0.34	1.78 ± 0.42	1.68 ± 0.42	1.48 ± 0.35	1.62 ± 0.39	1.75 ± 0.40	1.88 ± 0.44
Age, year	62.9 ± 13.4	63.9 ± 13.7	63.0 ± 13.4	62.5 ± 13.2	62.3 ± 13.2	62.1 ± 13.2	63.7 ± 13.5	62.0 ± 13.2	61.3 ± 12.9	61.5 ± 13.0
Female sex, no. (%)	3,574 (40.8)	959 (26.8)	770 (21.5)	797 (22.3)	1,048 (29.3)	3,580 (40.8)	925 (25.8)	978 (27.3)	929 (25.9)	748 (20.9)
Geographic region, no. (%)										
Western Europe/North America	1,544 (17.6)	567 (36.7)	394 (25.5)	302 (19.6)	281 (18.2)	1,544 (17.6)	381 (24.7)	439 (28.4)	447 (29.0)	277 (17.9)
Eastern/Central Europe/ Middle East	1,079 (12.3)	206 (19.1)	213 (19.7)	290 (26.9)	370 (34.3)	1,079 (12.3)	211 (19.6)	287 (26.6)	322 (29.8)	259 (24.0)
Africa	587 (6.70)	278 (47.4)	126 (21.5)	90 (15.3)	93 (15.8)	587 (6.70)	213 (36.3)	192 (32.7)	118 (20.1)	64 (10.9)
China	3,891 (44.4)	728 (18.7)	836 (21.5)	1,053 (27.1)	1,274 (32.7)	3,891 (44.4)	832 (21.4)	1,089 (28.0)	1,051 (27.0)	919 (23.6)
South East Asia	615 (7.02)	155 (25.2)	123 (20.0)	125 (20.3)	212 (34.5)	615 (7.01)	204 (33.2)	214 (34.8)	126 (20.5)	71 (11.5)
South America	1,045 (11.9)	280 (26.8)	219 (21.0)	205 (19.6)	341 (32.6)	1,051 (12.0)	234 (22.3)	260 (24.7)	288 (27.4)	269 (25.6)
Stroke type, no. (%)										
Ischemic	6,805 (77.7)	1,710 (77.4)	1,537 (81.0)	1,646 (80.0)	1,912 (74.7)	—	—	—	—	—
ICH	1,919 (21.9)	499 (22.6)	361 (19.0)	411 (20.0)	648 (25.3)	—	—	—	—	—
Hypertension, no. (%)	5,243 (59.8)	1,363 (61.6)	1,134 (59.3)	1,192 (57.7)	1,554 (60.4)	3,299 (37.6)	974 (46.9)	998 (40.2)	988 (42.0)	856 (46.0)
Blood pressure, mm Hg										
Systolic	148 ± 21.2	149 ± 22.0	147 ± 20.8	148 ± 20.5	149 ± 21.3	133 ± 18.5	133 ± 19.1	133 ± 18.3	133 ± 17.8	134 ± 18.7
Diastolic	86.5 ± 12.3	87.0 ± 13.2	85.4 ± 12.1	86.5 ± 11.8	86.9 ± 12.0	80.1 ± 10.7	79.3 ± 11.2	79.8 ± 10.5	80.3 ± 10.3	81.1 ± 10.6
Cholesterol, mmol/l										
HDL	1.15 ± 0.35	1.18 ± 0.37	1.16 ± 0.37	1.14 ± 0.34	1.12 ± 0.33	1.22 ± 0.37	1.22 ± 0.38	1.23 ± 0.38	1.21 ± 0.35	1.20 ± 0.37
LDL	2.97 ± 1.01	3.03 ± 1.08	2.96 ± 0.98	2.99 ± 0.99	2.91 ± 0.98	2.98 ± 0.96	2.98 ± 1.01	3.04 ± 0.97	2.97 ± 0.94	2.90 ± 0.92
Diabetes mellitus, no. (%)	1,486 (17.0)	355 (16.0)	315 (16.5)	359 (17.4)	457 (17.8)	1,108 (12.6)	283 (13.6)	304 (12.3)	303 (12.9)	218 (11.7)
AFIB/atrial flutter, no. (%)	936 (10.7)	301 (13.6)	210 (11.0)	170 (8.23)	255 (9.92)	270 (3.08)	74 (3.57)	69 (2.78)	61 (2.59)	66 (3.55)
Diuretic preadmission, no. (%)	1,132 (12.9)	298 (13.5)	260 (13.6)	229 (11.1)	345 (13.4)	782 (8.92)	194 (9.35)	185 (7.46)	204 (8.68)	199 (10.7)
Diuretic in hospital, no. (%)	1,994 (22.8)	537 (24.3)	418 (21.9)	441 (21.4)	598 (23.3)	352 (12.8)	73 (11.3)	80 (10.2)	95 (13.7)	104 (16.3)
Current smoker, no. (%)	2,623 (29.9)	610 (27.6)	591 (30.9)	666 (32.3)	756 (29.4)	1,850 (21.1)	429 (20.7)	522 (21.0)	510 (21.7)	389 (20.9)

Abbreviations: AFIB, atrial fibrillation; HDL, high-density lipoprotein; ICH, intracerebral hemorrhage; LDL, low-density lipoprotein.

### Estimated sodium excretion (quartiles) and blood pressure

**Figure 2.**
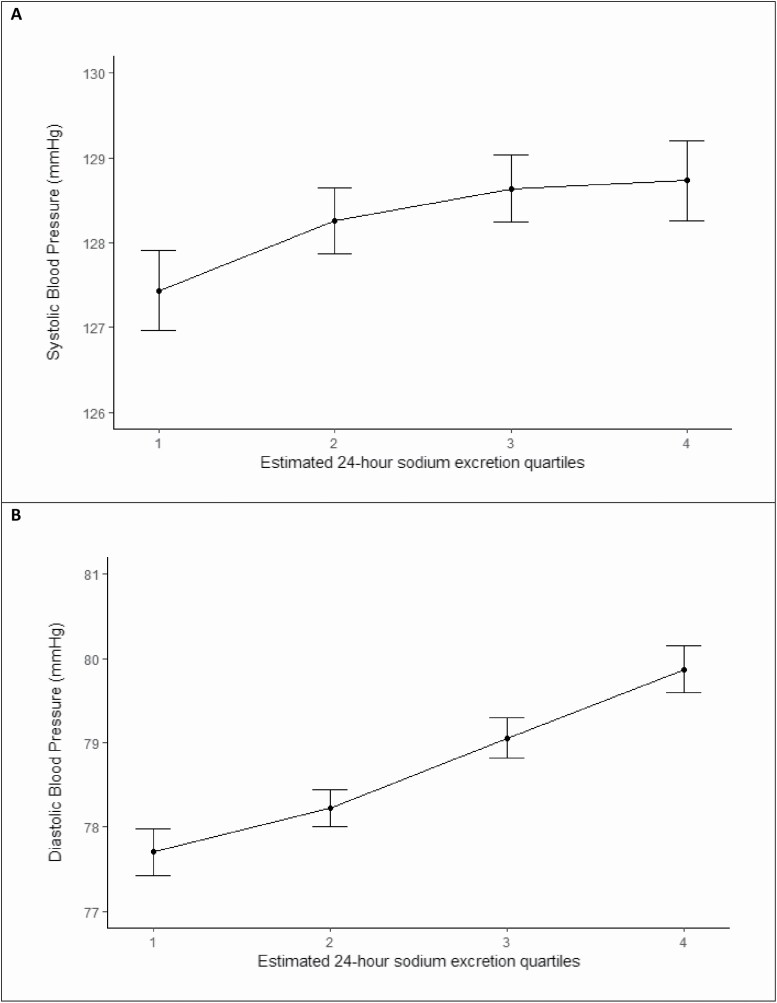
Mean systolic and diastolic blood pressure by sodium quartile (controls excluding baseline hypertension and prehospital diuretic use).

Figure 2 reports the association of urinary sodium excretion and blood pressure among controls not receiving antihypertensive therapy or diuretics and indicates a graded increase in blood pressure with increasing sodium intake. For each 1-g increment in estimated sodium excretion, there was an increment of 1.01 mm Hg in systolic blood pressure (*P* < 0.001) and an increment of 0.48 mm Hg in diastolic blood pressure (*P* < 0.001).

### Estimated sodium excretion (quartiles) and risk of stroke and stroke subtypes

**Figure 3.**
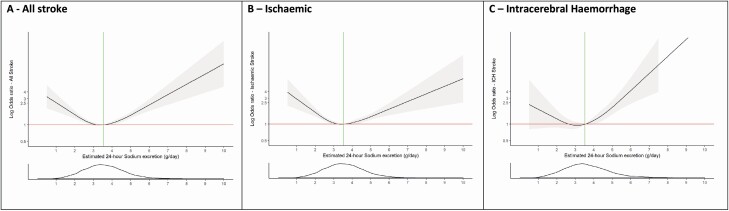
Association of estimated 24-hour sodium excretion (Tanaka) with risk of stroke and pathological stroke subtypes. Panel **a** shows a restricted cubic spline of the association between estimated 24-hour sodium excretion and risk of all stroke. Panel **b** shows a restricted cubic spline of the association between estimated 24-hour sodium excretion and risk of ischemic stroke. Panel **c** shows a restricted cubic spline of the association between estimated 24-hour sodium excretion and risk of intracerebral hemorrhage. All plots were adjusted for age, BMI, education level, alcohol intake, diabetes at baseline, atrial fibrillation/flutter at baseline, smoking, and physical activity level. The gray ribbons indicate 95% confidence interval. The green lines represent the median value for each population. The distribution of the exposure (sodium excretion) is plotted below each spline. Abbreviation: BMI, body mass index.

**Figure 4.**
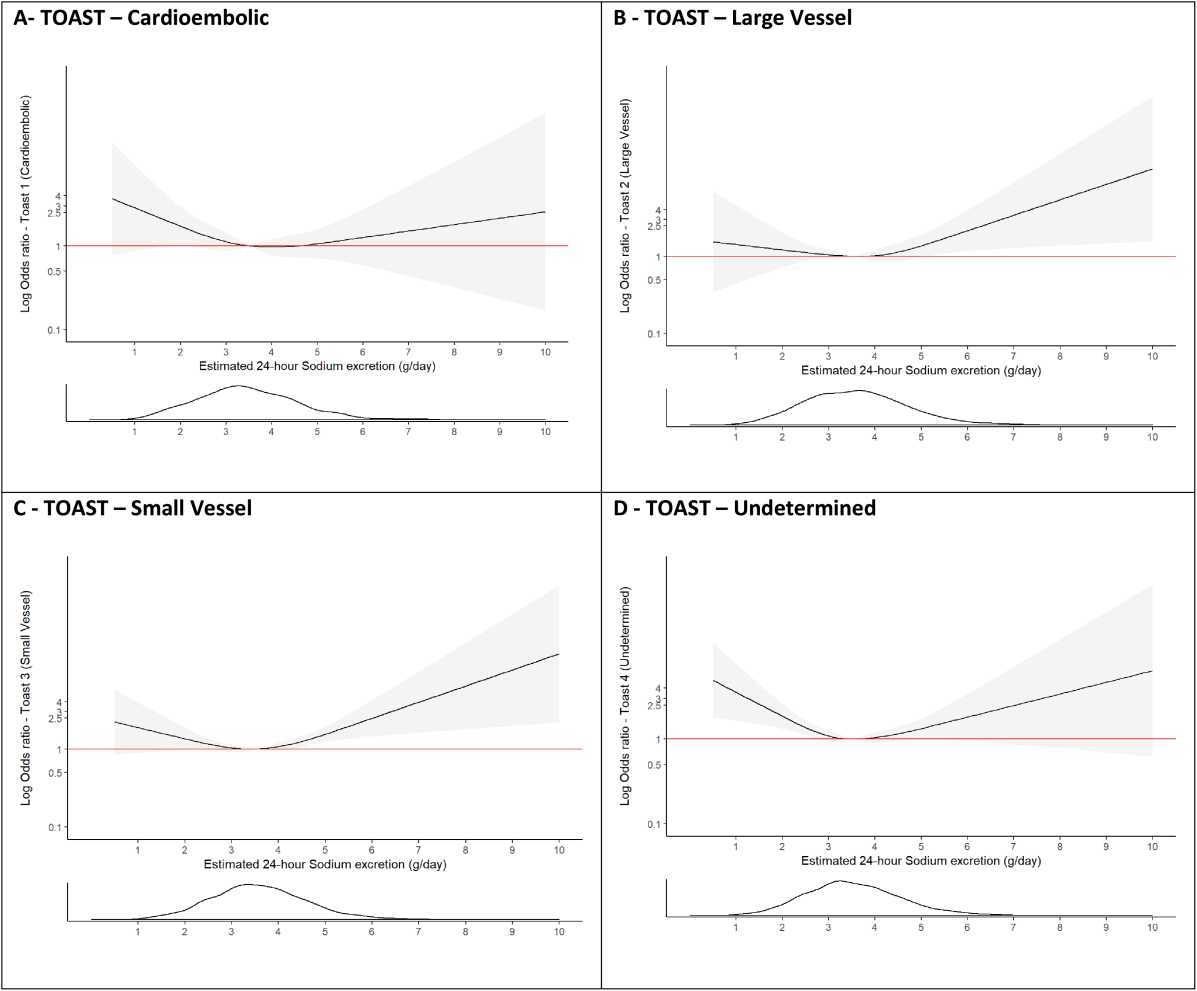
Association of estimated sodium excretion (Tanaka) and risk of ischemic stroke subtypes (TOAST classification). Panel **a** shows a restricted cubic spline of the association between estimated 24-hour sodium excretion and cardioembolic stroke (TOAST 1). Panel **b** shows a restricted cubic spline of the association between estimated 24-hour sodium excretion and large vessel stroke (TOAST 2). Panel **c** shows a restricted cubic spline of the association between estimated 24-hour sodium excretion and small vessel stroke (TOAST 3). Panel **d** shows a restricted cubic spline of the association between estimated 24-hour sodium excretion and stroke of undetermined cause (TOAST 4). All plots were adjusted for age, BMI, education level, alcohol intake, diabetes at baseline, atrial fibrillation/flutter at baseline, smoking, and physical activity level. The gray ribbons indicate 95% confidence interval. The distribution of the exposure (potassium excretion) is plotted below each spline. Abbreviation: BMI, body mass index.

Compared with Q2 (sodium excretion of 2.8–3.5 g/day [reference]), Q1 (odds ratio [OR] 1.39; 95% confidence interval [CI], 1.26–1.53, sodium excretion <2.8 g/day), and Q4 (OR 1.81; 95% CI, 1.65–2.00, sodium excretion >4.26 g/day) were associated with significant increases in the risk of all stroke (Table 2). The highest quartile (Q4 >4.26 g/day) was more strongly associated with ICH (OR 2.38; 95% CI, 1.93–2.92) than ischemic stroke (OR 1.67; 95% CI, 1.50–1.87) (*P* < 0.001). Sodium excretion <2.8 g/day was significantly associated with both ischemic stroke (OR 1.36; 95% CI, 1.22–1.52) and ICH (OR 1.62; 95% CI, 1.32–1.99) (Figure 3 and Supplementary Tables S5 and S6 online). The association of high sodium excretion (>4.26 g/day) and stroke remained significant after adjustment for blood pressure and prior history of hypertension (OR 2.35; 95% CI, 2.08–2.65). Within ischemic stroke subtypes, the association of high sodium intake was significant for small vessel and large vessel ischemic stroke, but not significant for cardioembolic stroke (Figure 4).

**Table 2.** Association of estimated 24-hour sodium excretion quartiles and risk of stroke

	Estimated sodium excretion			
	<2.8 g/day	2.8–3.5 g/day	3.5–4.26 g/day	>4.26 g/day
	(*N* = 4,751)	(*N* = 4,750)	(*N* = 4,750)	(*N* = 4,750)
Analysis—odds ratio (95% CI)				
Univariate analysis^a^	1.40 (1.28–1.52)	1.00	1.15 (1.06–1.25)	1.84 (1.69–2.01)
Multivariate analysis				
Analysis including age and BMI	1.41 (1.29–1.54)	1.00	1.14 (1.05–1.25)	1.86 (1.70–2.03)
Primary analysis^b^	1.39 (1.26–1.53)	1.00	1.13 (1.03–1.24)	1.81 (1.65–2.00)
Analysis including dietary score and potassium^c^	1.24 (1.12–1.37)	1.00	1.28 (1.16–1.41)	2.49 (2.24–2.77)
Analysis including HTN and medications which modify sodium excretion^d^	1.16 (1.03–1.30)	1.00	1.22 (1.09–1.36)	2.35 (2.08–2.65)
Sensitivity analysis				
Primary analysis excluding MRC >2	1.37 (1.18–1.58)	1.00	1.08 (0.95–1.23)	1.64 (1.42–1.88)
Primary analysis excluding urine collection >48 hours	1.28 (1.12–1.47)	1.00	1.18 (1.03–1.34)	1.91 (1.67–2.18)

Urine collection from time of stroke onset to time of urine collection. Abbreviations: ACE, angiotensin-converting enzyme inhibitors (ACE inhibitors); BMI, body mass index; CI, confidence interval; HTN, hypertension; MRC, modified Rankin scale.

^a^The univariate analysis was performed using the logistic regression model.

^b^The primary model included age, BMI, education level, alcohol, diabetes at baseline, atrial fibrillation/flutter at baseline, smoking and physical activity level.

^c^Dietary score was the alternative healthy eating index (AHEI).

^d^Hypertension variables hypertension status, systolic blood pressure, and diastolic blood pressure. We adjusted for prehospital ACE inhibitor, angiotensin receptor blocker, and diuretic use.

### Estimated potassium excretion and risk of stroke and stroke subtypes

**Figure 5.**
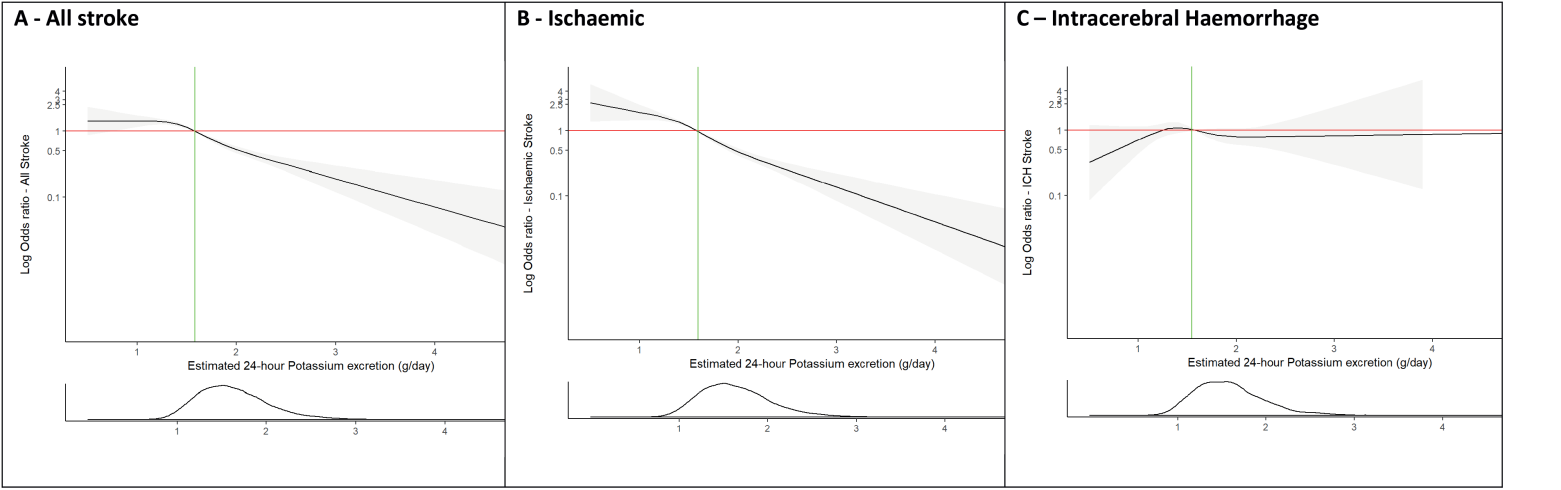
Association of estimated 24-hour potassium excretion (Tanaka) with risk of stroke and pathological stroke subtypes. Panel **a** shows a restricted cubic spline of the association between estimated 24-hour potassium excretion and risk of all stroke. Panel **b** shows a restricted cubic spline of the association between estimated 24-hour potassium excretion and risk of ischemic stroke. Panel **c** shows a restricted cubic spline of the association between estimated 24-hour potassium excretion and risk of intracerebral hemorrhage. All plots were adjusted for age, BMI, education level, alcohol intake, diabetes at baseline, atrial fibrillation/flutter at baseline, smoking, and physical activity level. The gray ribbons indicate 95% confidence interval. The green lines represent the median value for each population. The distribution of the exposure (sodium excretion) is plotted below each spline. Abbreviation: BMI, body mass index.

Compared with Q1 (potassium excretion of <1.34 g/day [reference]), Q2 (OR 0.83; 95% CI, 0.76–0.92, potassium excretion 1.34–1.58 g/day), Q3 (OR 0.68; 95% CI, 0.62–0.75, potassium excretion 1.58–1.86 g/day), and Q4 (OR 0.46; 95% CI, 0.41–0.51, potassium excretion >1.86 g/day) were all associated with a significant lower risk of all stroke, which was largely related to the association of potassium excretion with ischemic stroke and there was no significant association with ICH (Table 3, Figure 5).

**Table 3.** Association of estimated 24-hour potassium excretion quartiles and risk of stroke

	Estimated potassium excretion quartiles			
	Quartile 1	Quartile 2	Quartile 3	Quartile 4
	<1.34 g/day	1.34–1.58 g/day	1.58–1.86 g/day	>1.86 g/day
	(*N* = 4,817)	(*N* = 4,816)	(*N* = 4,817)	(*N* = 4,816)
Analysis—odds ratio (95% CI)				
Univariate analysis^a^	1.00	0.80 (0.73–0.87)	0.67 (0.61–0.73)	0.43 (0.39–0.47)
Multivariate analysis				
Analysis including age and BMI	1.00	0.80 (0.73–0.88)	0.64 (0.59–0.71)	0.42 (0.38–0.46)
Primary analysis^b^	1.00	0.83 (0.76–0.92)	0.68 (0.62–0.75)	0.46 (0.41–0.51)
Analysis including dietary score and sodium^c^	1.00	0.75 (0.68–0.83)	0.56 (0.51–0.63)	0.33 (0.29–0.37)
Analysis including HTN and medications which modify potassium excretion^d^	1.00	0.76 (0.68–0.86)	0.57 (0.51–0.65)	0.33 (0.29–0.38)
Sensitivity analysis				
Primary analysis excluding MRC >2	1.00	0.70 (0.60–0.81)	0.48 (0.41–0.56)	0.24 (0.20–0.28)
Primary analysis excluding urine collection >48 hours	1.00	0.87 (0.76–0.99)	0.78 (0.68–0.89)	0.67 (0.58–0.77)

Urine collection from time of stroke onset to time of urine collection. Abbreviations: ACE, angiotensin-converting enzyme inhibitors (ACE inhibitors); BMI, body mass index; CI, confidence interval; HTN, hypertension; MRC, modified Rankin scale.

^a^The univariate analysis was performed using the logistic regression model.

^b^The primary model included age, BMI, education level, alcohol, diabetes at baseline, atrial fibrillation/flutter at baseline, smoking, and physical activity level.

^c^Dietary score was the alternative healthy eating index (AHEI).

^d^Hypertension variables hypertension status, systolic blood pressure, and diastolic blood pressure. We adjusted for prehospital ACE inhibitor, angiotensin receptor blocker, and diuretic use.

### Joint urinary sodium and potassium excretion and risk of stroke

For all stroke, compared with a joint reference category of moderate sodium excretion (2.8–3.5 g/day) and high potassium intake (≥1.58 g/day) (lowest risk category), all other categories were associated with an increased risk of stroke, with sodium excretion >4.26 g/day and potassium excretion <1.58 g/day reporting the highest magnitude of risk (OR 4.17; 95% CI, 3.51–4.96) (Table 4, Supplementary Figures S1 and S2 online). The magnitude of association is reduced for both low (<2.8 g/day) and high (>4.26 g/day) sodium excretion when potassium excretion is high (≥1.58 g/day) (*P* < 0.001 for interaction) (Table 4).

**Table 4.** Association of joint urinary sodium and potassium excretion with stroke

	Joint association of urinary sodium and potassium excretion with stroke				
	Quartile 1	Quartile 2	Quartile 3	Quartile 4	
	<2.8 g/day	2.8–3.5 g/day	3.5–4.26 g/day	>4.26 g/day	
Potassium less than the median (<1.58 g/day)	OR_Joint_ 2.10 (1.89–2.50)	OR_Joint_ 1.94 (1.69–2.24)	OR_Joint_ 2.62 (2.26–3.05)	OR_Joint_ 4.17 (3.51–4.96)	*P* for interaction
	(*n* = 3,176)	(*n* = 2,600)	(*n* = 2,217)	(*n* = 1,471)	<0.001
Potassium greater than or equal to the median (≥1.58 g/day)	OR_Joint_ 1.69 (1.44–1.98)	Ref 1.0	OR_Joint_ 1.10 (0.95–1.26)	OR_Joint_ 2.26 (1.97–2.59)	
	(*n* = 1,575)	(*n* = 2,149)	(*n* = 2,530)	(*n* = 3,278)	

The primary model included age, BMI, education level, alcohol, diabetes at baseline, atrial fibrillation/flutter at baseline, smoking, and physical activity level. Abbreviations: BMI, body mass index; OR, odds ratio.

### Subgroup and sensitivity analysis of the associations between sodium and potassium excretion and risk of stroke

There was a significantly increased risk of stroke for Q4 (>4.26 g/day) vs. Q2 (2.8–3.5 g/day) in participants with a body mass index less than or equal to 30 (OR 1.92; 95% CI, 1.72–2.15) compared with participants with a body mass index greater than 30 (OR 1.35; 95% CI, 0.81–2.26) (*P* = 0.009 for interaction). The association of high sodium excretion with stroke (>4.26 g/day) vs. Q2 (2.8–3.5 g/day) was significant for European (OR 1.34; 95% CI, 1.11–1.63), Chinese (OR 1.85; 95% CI, 1.59–2.14), Other Asian (OR 10.83 (6.35–18.48), Latin American (OR 1.61; 95% CI, 1.23–2.13), Black African (OR 1.95; 95% CI, 1.10–3.47), and “Other” African ethnicity (OR 1.96; 95% CI, 1.12–3.43). The association of low sodium excretion with stroke (<2.8 g/day) vs. Q2 (2.8–3.5 g/day) was significant for European (OR 1.57; 95% CI, 1.30–1.89), Arab (OR 2.51; 95% CI, 1.13–5.56), Latin American (OR 1.40; 95% CI, 1.06–1.87), Black African (OR 2.21; 95% CI, 1.37–3.55), and Other ethnicity (OR 2.21; 95% CI, 1.43–3.41) (*P* < 0.001 for interaction). The associations for both high sodium excretion (OR 1.46; 95% CI, 0.77–2.76) and low sodium excretion (OR 1.55; 95% CI, 0.71–3.41) compared with Q2 (2.8–3.5 g/day) were nonsignificant for participants with diuretic use at baseline (*P* = 0.1505 for interaction). Sex, age, or baseline hypertension status did not alter the association significantly between both high and low estimated sodium excretion and stroke (Supplementary Table S7 online).

The exclusion of cases with modified Rankin scale greater than 2, and the exclusion of cases with urine collected greater than 48 hours after symptom onset did not materially alter findings (Tables 2 and 3).

We repeated all analyses with the Kawasaki formula, urinary sodium/creatinine ratio, and urinary sodium, which revealed consistent patterns of association, but, as expected, different thresholds of sodium and potassium excretion (g/day) were associated with stroke risk (Supplementary Figures S3–S6 online).

## DISCUSSION

In this large, international, case–control study, we report an overall J-shaped association between sodium intake and stroke risk, with the lowest risk at moderate sodium intake (2.8–3.5 g/day), employing estimated 24-hour urinary excretion of sodium as a surrogate for intake. The association of high sodium intake was stronger for ICH compared with ischemic stroke and within ischemic stroke subtypes, was significant for small vessel and large vessel ischemic stroke, but not significant for cardioembolic stroke. The association between estimated potassium excretion and risk of ischemic stroke was inverse and linear, but not significant for ICH. The magnitude of association for both low (<2.8 g/day) and high (>4.26 g/day) sodium excretion was diminished in those with high potassium intake (≥1.58 g/day) (*P* < 0.001 for interaction). In our analysis of combined categories of sodium and potassium intake, the category of highest potassium intake (≥1.58 g/day) and moderate sodium intake (2.8–3.5 g/day) was associated with the lowest risk of stroke.

Most national and international guidelines recommend low sodium intake in the entire population, for stroke prevention (e.g., WHO recommend an intake of <2.0 g/day). Primarily, the target of low sodium is based on the short-term Phase IIa DASH-Sodium trial which reported a blood pressure reduction when reducing sodium intake to less than 1.5 g/day by providing all meals to the participants,^24^ the longer-term trials (TONE and TOHP-2) which achieved mean sodium intakes of 2.3 and 3.11 g/day (despite targeting a sodium intake of 1.8 g/day or lower) through intense dietary counseling.^25,26^ While a target of <2.0 g/day can be achieved in a highly controlled environment, we report that a very low proportion of the population consume a low sodium intake, and an even lower proportion consume a combined low sodium and high potassium intake. Our findings are consistent with other epidemiologic studies, and support the contention that a lower limit of sodium intake exists among free-living populations due to neurohormonal control mechanisms that autoregulate the consumption of sodium.^27^ Activation of the renin–angiotensin–aldosterone system occurs when sodium intake falls below approximately 3.0 g/day. An analysis of the HOPE study reported a positive association between higher quintiles of plasma renin activity and cardiovascular outcomes including stroke,^28^ and consistently, the relative risk for the highest quintile of plasma renin activity was 1.43, identical to our estimate for the stroke risk associated with sodium excretion <2.8 g/day. In addition, we report a different pattern of association between sodium intake and blood pressure (positive and monotonic) compared with the pattern of association with stroke risk (J-shaped). These patterns have also been reported in several recent large cohort studies,^7,29^ and challenge assumptions that underpin current guidelines (i.e., that all reductions in blood pressure will reduce stroke, regardless of baseline sodium intake level).^9^ Our findings do however, support public health interventions to reduce sodium intake among populations with high sodium intake and support transitioning populations from high to moderate sodium intake in order to reduce stroke.^30^ Our data suggest that the risk associated with a higher sodium intake may be greater in regions outside of Europe and North America.

Our data also provide important insights into the anticipated effects of reducing high sodium intake on patterns of stroke and its subtypes; reducing high sodium intake is likely to have a greater effect on reducing ICH than ischemic stroke, but is nevertheless expected to also reduce ischemic stroke, and thereby the global burden of all stroke. In ecological studies of stroke incidence in China, for example, population-level reductions in high sodium intake parallel reductions in stroke incidence, and are more marked for ICH than ischemic stroke.^31^ In the INTERSTROKE study, about 40% of the control population were consuming higher sodium intake in a range associated with stroke risk, supporting a targeted-population approach to sodium reduction, rather than a population-wide approach.

Our analyses also suggest that increases in potassium intake may be of comparable, or greater, importance to stroke prevention than reductions in sodium intake.^12,16,17^ This finding is consistent with reports that high potassium intake is a marker of a healthy diet, i.e., rich in fruit and vegetables.^32^

A recent analysis of the PURE cohort study reported that the combination of moderate sodium intake and high potassium intake was associated with the lowest cardiovascular risk, and our analyses are further evidence that such a combined target may be optimal.^12^ A cluster randomized controlled trial reported significant reductions in cardiovascular risk with potassium salt substitution.^33^ Potassium salt substitution not only increased potassium intake but also reduced high sodium intake (but not to low intake levels).^33^ An ongoing large cluster randomized controlled trial in China is currently evaluating potassium salt substitutes for prevention of stroke.^34^ Our analyses, and those of other studies, raise major concerns about the feasibility of increasing potassium intake, while simultaneously achieving low sodium intake. They suggest that populations should target moderate sodium intake and high potassium intake as the optimal balance, as the former is expected to make the latter more achievable.

Measurement of sodium and potassium intake is a major challenge, and there is no “gold” standard for estimating usual sodium and potassium intake.^35^ The reference standard,^36^ repeated 24-hour urinary collections, is impractical in large epidemiologic studies and would invariably lead to the exclusion of a substantial proportion of participants and a biased sample. In our study, the mean intake of sodium in the control group was 3.54 g/day, which is close to the mean intake reported by the Global Burden of Diseases Nutrition and Chronic Diseases Expert Group (3.95 g/day) and the PURE study (4.9 g/day).^7,37^ In the PURE study, a fasting morning urine sample was collected and the Kawasaki formula was used to estimate sodium excretion. In contrast, we collected a random urine sample and used the Tanaka formula to estimate sodium excretion. A validation study of 1,083 participants from the PURE study showed a similar differences between mean sodium estimated using the Kawasaki equation and Tanaka equation.^21^ Importantly, however, irrespective of the method we employed in INTERSTROKE, which included urinary sodium/creatinine ratio, Tanaka formula or Kawasaki formula, the J-shaped pattern of association were consistent among all analyses, and median intake levels are associated with lowest cardiovascular risk. Collectively, despite the study-by-study variation in methods of estimating sodium intake, there is a remarkable consistency in findings from large epidemiologic studies that the optimal range of sodium intake resides within a range between 2.7 and 5.0 g/day. This is also the range identified in a meta-analysis of prospective cohort studies published before 2014,^9^ by Graudal *et al.*, and consistent with prospective cohort studies reported since then, including PURE,^12^ CRIC,^38^ and PREVEND^39^ studies.

The case–control design has inherent limitations, including sampling bias (selection of cases and controls) and measurement bias (recall bias). Sensitivity analysis by control type (community vs. hospital) did not alter our findings and estimated urinary sodium excretion is an objective lab measurement and not susceptible to recall bias. Another limitation is the potential acute effects of stroke on excretion of sodium intake, particularly the change in oral intake and use of intravenous fluids in those with severe stroke. To address this issue, we performed several sensitivity analyses by excluding patients with a modified Rankin score greater than 2 as these patients are likely have received intravenous fluids and enteric feeding due to their disability. Excluding these patients did not materially alter our findings. In addition, increasing time from admission to urinary sample measurement may reduce the correlation of usual (prestroke) diet with urinary estimate. Confining the analyses to those with early urine collections (<48 hours) did not alter conclusions.

A key strength of the INTERSTROKE study was the availability of neuroimaging to classify all cases of stroke. INTERSTROKE is the only large study which reliably examines whether the associations of sodium and potassium differs between ischemic stroke, ischemic stroke subtypes, and ICH. Obtaining such information from cohort studies is impractical. The diverse international population included in our results are widely generalizable.

In conclusion, an estimated sodium excretion <2.8 and >4.26 g/day are both associated with an increased risk of stroke (reference 2.8–3.5 g/day). An estimated potassium excretion of greater than 1.34 g/day was associated with a reduced risk of stroke (reference <1.34 g/day). In the absence of large randomized controlled trials, the collective information from observational data on over 300,000 individuals suggests that the optimal intake of sodium is a moderate level combined with high potassium intake.

## Supplementary Material

hpaa176_suppl_Supplementary_Appendix
