# Supplementary material for: Urinary Sodium and Potassium, and Risk of Ischemic and Hemorrhagic Stroke (INTERSTROKE): A Case–Control Study
Source: Am J Hypertens. 2020 Nov 16;34(4):414–25. doi: 10.1093/ajh/hpaa176 (PMC8057138; doi:10.1093/ajh/hpaa176)

Contents

[Supplementary Table 1 – Guidance to Sites for Selection of Controls 2](#_Toc50749267)

[Supplementary Table 2 – Risk factors collected in INTERSTROKE 3](#_Toc50749268)

[Supplementary Table 3 – Characteristics of the Study Participants at Baseline, According to Estimated Sodium Excretion (Unconditional Analysis) 4](#_Toc50749269)

[Supplementary Table 4 – Characteristics of the Study Participants at Baseline, According to Estimated Potassium Excretion (Conditional Analysis) 6](#_Toc50749270)

[Supplementary Table 5 - Association of estimated 24-hour sodium excretion and risk of ***ischaemic*** stroke 8](#_Toc50749271)

[Supplementary Table 6 - Association of estimated 24-hour sodium excretion and risk of ***intracerebral haemorrhage*** 9](#_Toc50749272)

[Supplementary Table 7 – Multivariate (Conditional) Association Between Estimated 24-Hour Urinary Sodium Excretion and Stroke in Subgroups 10](#_Toc50749273)

[Supplementary Figure 1 – Association of Estimated 24-hr sodium excretion with risk of all stroke (Below Median Potassium) 11](#_Toc50749274)

[Supplementary Figure 2 – Association of Estimated 24-hr sodium excretion with risk of all stroke (Above Median Potassium) 12](#_Toc50749275)

[Supplementary Figure 3 – Association of Estimated 24-hr sodium excretion (Kawasaki) with risk of all stroke 13](#_Toc50749276)

[Supplementary Figure 4 – Association of Estimated 24-hour sodium excretion (Kawasaki) with risk of Ischaemic stroke 14](#_Toc50749277)

[Supplementary Figure 5 – Association of Estimated 24-hour sodium excretion (Kawasaki) with risk of ICH 15](#_Toc50749278)

[Supplementary Figure 6 – Association of Estimated 24-hour sodium excretion (Urinary Sodium Creatinine Ratio) with risk of All Stroke 16](#_Toc50749279)

[Supplementary Figure 7 – Association of Estimated 24-hour sodium excretion (Urinary Sodium Creatinine Ratio) with risk of Ischaemic stroke 17](#_Toc50749280)

[Supplementary Figure 8 – Association of Estimated 24-hour sodium excretion (Urinary Sodium Creatinine Ratio) with risk of ICH 18](#_Toc50749281)

## Supplementary Table 1 – Guidance to Sites for Selection of Controls

| **Selection of Controls for INTERSTROKE Study** |
| --- |
| **Controls:** 1. Community-based control. 2. Relative of a patient from a non-cardiac ward. 3. Unrelated (not first degree relative) visitor of any patient. 4. Patients attending the hospital or outpatient clinic**:**  **Preferred controls from hospital settings:** Patients attending the hospital or outpatients clinic for the following reasons: 4.1 Refraction and cataracts (excluding those presenting with acute visual loss). 4.2 Physical check-up. 4.3 Routine pap smear. 4.4 Routine breast exam. 4.5 Elective minor surgery for conditions that are not obviously related to stroke or its risk factors. 4.6 Elective orthopaedic surgery.  **Acceptable controls from hospital settings:** Patients attending the hospital or outpatients clinic for the following reasons: 4.7 Outpatient fractures. 4.8 Arthritic complaints. 4.9 Plastic surgery. 4.10 Haemorrhoids, hernias, hydroceles. 4.11 Routine colon cancer screening. 4.12 Endoscopy. 4.13 Minor dermatological disorders. |

## Supplementary Table 2 – Risk factors collected in INTERSTROKE

| **Risk factors collected in INTERSTROKE** |
| --- |
| - Demographic features   - Age   - Sex   - Education level - Cardiovascular risk factors   - Prior history of hypertension   - Diabetes mellitus   - Atrial fibrillation - Lifestyle risk factors   - Alcohol intake   - Tobacco use   - Physical activity   - Diet (food frequency questionnaire). |

##

## Supplementary Table 3 – Characteristics of the Study Participants at Baseline, According to Estimated Sodium Excretion (Unconditional Analysis)

|  | **Characteristic** | | **Case** | | | | | **Control** | | | | |
| --- | --- | --- | --- | --- | --- | --- | --- | --- | --- | --- | --- | --- |
|  |  |  | **Estimated Sodium Excretion** | | | | | | | | | |
|  |  |  | All | <2.8 g/day | 2.8-3.5 g/day | 3.5-4.3 g/day | >4.3 g/day | All | <2.8 g/day | 2.8-3.5 g/day | 3.5-4.3 g/day | >4.3 g/day |
|  |  |  | (N=10511) | (N=2392) | (N=2010) | (N=2155) | (N=2718) | (N=10533) | (N=2359) | (N=2740) | (N=2595) | (N=2032) |
|  | Estimated excretion - g/day | |  |  |  |  |  |  |  |  |  |  |
|  |  | Sodium | 3.68±1.28 | 2.22±0.44 | 3.19±0.20 | 3.89±0.21 | 5.18±1.00 | 3.53±1.04 | 2.32±0.41 | 3.19±0.20 | 3.88±0.21 | 4.94±0.92 |
|  |  | Potassium | 1.58±0.38 | 1.43±0.30 | 1.49±0.33 | 1.56±0.34 | 1.78±0.42 | 1.68±0.42 | 1.48±0.35 | 1.63±0.39 | 1.75±0.40 | 1.89±0.44 |
|  | Age - yr | | 62.9±13.7 | 63.9±13.8 | 62.9±13.4 | 62.4±13.4 | 62.3±13.5 | 62.1±13.4 | 63.7±13.7 | 62.0±13.2 | 61.2±13.2 | 61.6±13.0 |
|  | Female Sex - no. (%) | | 4376 (41.6%) | 1037 (43.4%) | 806 (40.1%) | 828 (38.4%) | 1111 (40.9%) | 4386 (41.6%) | 1055 (44.7%) | 1106 (40.4%) | 1064 (41.0%) | 820 (40.4%) |
|  | Geographic region - no. (%) | |  |  |  |  |  |  |  |  |  |  |
|  |  | Western Europe / North America | 1915 (18.2) | 602 (25.2) | 419 (20.8) | 322 (14.9) | 301 (11.1) | 1917 (18.2) | 436 (18.5) | 508 (18.5) | 511 (19.7) | 327 (16.1) |
|  |  | Eastern / Central Europe / Middle East | 1355 (12.9) | 224 (9.36) | 227 (11.3) | 301 (14.0) | 390 (14.3) | 1355 (12.9) | 237 (10.0) | 313 (11.4) | 340 (13.1) | 278 (13.7) |
|  |  | Africa | 971 (9.24) | 336 (14.0) | 148 (7.36) | 104 (4.83) | 110 (4.05) | 975 (9.26) | 278 (11.8) | 232 (8.47) | 159 (6.13) | 84 (4.13) |
|  |  | China | 3976 (37.8) | 737 (30.8) | 842 (41.9) | 1062 (49.3) | 1281 (47.1) | 3976 (37.7) | 849 (36.0) | 1104 (40.3) | 1063 (41.0) | 923 (45.4) |
|  |  | South East Asia | 833 (7.93) | 168 (7.02) | 129 (6.42) | 129 (5.99) | 222 (8.17) | 833 (7.91) | 257 (10.9) | 260 (9.49) | 161 (6.20) | 101 (4.97) |
|  |  | South America | 1461 (13.9) | 325 (13.6) | 245 (12.2) | 237 (11.0) | 414 (15.2) | 1477 (14.0) | 302 (12.8) | 323 (11.8) | 361 (13.9) | 319 (15.7) |
|  | Stroke type - no. (%) | |  |  |  |  |  |  | . | . | . | . |
|  |  | Ischemic | 8217 (78.5) | 1849 (77.5) | 1620 (81.1) | 1710 (79.6) | 2019 (74.6) | - | - | - | - | - |
|  |  | ICH | 2250 (21.5) | 537 (22.5) | 377 (18.9) | 437 (20.4) | 688 (25.4) | - | - | - | - | - |
|  | Hypertension - no. (%) | | 6353 (60.4) | 1472 (61.5) | 1192 (59.3) | 1244 (57.7) | 1645 (60.5) | 4071 (38.7) | 990 (42.0) | 957 (34.9) | 934 (36.0) | 826 (40.7) |
|  | Blood pressure - mm Hg | |  |  |  |  |  |  |  |  |  |  |
|  |  | Systolic | 149±21.6 | 150±22.0 | 147±20.8 | 148±20.9 | 149±21.4 | 133±18.6 | 133±19.5 | 133±18.4 | 133±17.9 | 134±18.7 |
|  |  | Diastolic | 86.5±12.5 | 87.0±13.2 | 85.4±12.1 | 86.6±11.9 | 87.0±12.2 | 79.8±10.8 | 79.2±11.4 | 79.7±10.6 | 80.1±10.3 | 81.0±10.6 |
|  | Cholesterol - mmol/litre | |  |  |  |  |  |  |  |  |  |  |
|  |  | HDL | 1.15±0.35 | 1.17±0.37 | 1.15±0.36 | 1.14±0.34 | 1.12±0.33 | 1.22±0.38 | 1.22±0.39 | 1.23±0.38 | 1.22±0.36 | 1.20±0.37 |
|  |  | LDL | 2.97±1.03 | 3.02±1.09 | 2.94±0.98 | 2.98±0.98 | 2.91±0.98 | 2.96±0.97 | 2.96±1.02 | 3.03±0.97 | 2.96±0.94 | 2.89±0.92 |
|  | Diabetes mellitus - no. (%) | | 1876 (17.8) | 392 (16.4) | 338 (16.8) | 377 (17.5) | 484 (17.8) | 1400 (13.3) | 324 (13.7) | 350 (12.8) | 337 (13.0) | 251 (12.4) |
|  | AFIB/Atrial Flutter - no. (%) | | 1177 (11.2) | 321 (13.4) | 224 (11.1) | 176 (8.17) | 270 (9.93) | 342 (3.25) | 89 (3.77) | 78 (2.85) | 68 (2.62) | 70 (3.44) |
|  | Diuretic Pre-admission - no. (%) | | 1373 (13.1) | 320 (13.4) | 273 (13.6) | 242 (11.2) | 367 (13.5) | 988 (9.39) | 222 (9.41) | 216 (7.89) | 228 (8.79) | 231 (11.4) |
|  | Diuretic In Hospital - no. (%) | | 2440 (23.2) | 576 (24.1) | 431 (21.4) | 458 (21.3) | 626 (23.0) | 440 (12.3) | 88 (11.7) | 90 (10.3) | 102 (12.9) | 114 (15.9) |
|  | Current smoker - no. (%) | | 3044 (29.0) | 660 (27.6) | 618 (30.7) | 694 (32.2) | 785 (28.9) | 2123 (20.2) | 473 (20.1) | 560 (20.4) | 544 (21.0) | 415 (20.4) |

## Supplementary Table 4 – Characteristics of the Study Participants at Baseline, According to Estimated Potassium Excretion (Conditional Analysis)

|  | **Characteristic** | |  | **Case** | | | |  | **Control** | | | |
| --- | --- | --- | --- | --- | --- | --- | --- | --- | --- | --- | --- | --- |
|  |  |  | **Estimated Potassium Excretion** | | | | | | | | | |
|  |  |  | All | <1.34 g/day | 1.34-1.58 g/day | 1.58-1.86 g/day | >1.86 g/day | All | <1.34 g/day | 1.34-1.58 g/day | 1.58-1.86 g/day | >1.86 g/day |
|  |  |  | (N=8985) | (N=2240) | (N=1929) | (N=2079) | (N=2586) | (N=8991) | (N=2129) | (N=2519) | (N=2381) | (N=1879) |
|  | Estimated excretion - g/day | |  |  |  |  |  |  |  |  |  |  |
|  |  | Potassium | 1.57±0.38 | 1.43±0.30 | 1.49±0.33 | 1.56±0.34 | 1.78±0.42 | 1.68±0.42 | 1.48±0.35 | 1.62±0.39 | 1.75±0.40 | 1.89±0.44 |
|  |  | Sodium | 3.69±1.28 | 2.23±0.44 | 3.19±0.20 | 3.88±0.21 | 5.18±1.02 | 3.53±1.04 | 2.32±0.41 | 3.19±0.20 | 3.88±0.21 | 4.93±0.93 |
|  | Age - yr | | 62.9±13.4 | 63.9±13.7 | 63.0±13.4 | 62.5±13.2 | 62.3±13.2 | 62.1±13.2 | 63.7±13.6 | 62.1±13.2 | 61.3±12.9 | 61.5±12.9 |
|  | Female Sex - no. (%) | | 3688 (41.0%) | 973 (43.4%) | 777 (40.3%) | 803 (38.6%) | 1057 (40.9%) | 3694 (41.1%) | 954 (44.8%) | 997 (39.6%) | 947 (39.8%) | 755 (40.2%) |
|  | Geographic region - no. (%) | |  |  |  |  |  |  |  |  |  |  |
|  |  | Western Europe / North America | 1615 (18.0%) | 578 (25.8%) | 401 (20.8%) | 308 (14.8%) | 282 (10.9%) | 1615 (18.0%) | 393 (18.5%) | 455 (18.1%) | 457 (19.2%) | 284 (15.1%) |
|  |  | Eastern / Central Europe / Middle East | 1101 (12.3%) | 209 (9.33%) | 215 (11.1%) | 290 (13.9%) | 374 (14.5%) | 1101 (12.2%) | 215 (10.1%) | 289 (11.5%) | 325 (13.6%) | 261 (13.9%) |
|  |  | Africa | 657 (7.31%) | 286 (12.8%) | 130 (6.74%) | 92 (4.43%) | 96 (3.71%) | 657 (7.31%) | 235 (11.0%) | 205 (8.14%) | 128 (5.38%) | 68 (3.62%) |
|  |  | China | 3891 (43.3%) | 728 (32.5%) | 836 (43.3%) | 1053 (50.6%) | 1274 (49.3%) | 3891 (43.3%) | 832 (39.1%) | 1089 (43.2%) | 1051 (44.1%) | 919 (48.9%) |
|  |  | South East Asia | 643 (7.16%) | 158 (7.05%) | 125 (6.48%) | 126 (6.06%) | 214 (8.28%) | 643 (7.15%) | 212 (9.96%) | 218 (8.65%) | 129 (5.42%) | 73 (3.89%) |
|  |  | South America | 1078 (12.0%) | 281 (12.5%) | 222 (11.5%) | 210 (10.1%) | 346 (13.4%) | 1084 (12.1%) | 242 (11.4%) | 263 (10.4%) | 291 (12.2%) | 274 (14.6%) |
|  | Stroke type - no. (%) | |  |  |  |  |  |  |  |  |  |  |
|  |  | Ischemic | 6974 (77.9%) | 1733 (77.5%) | 1553 (81.1%) | 1657 (80.0%) | 1923 (74.7%) | - | - | - | - | - |
|  |  | ICH | 1973 (22.1%) | 502 (22.5%) | 363 (18.9%) | 414 (20.0%) | 652 (25.3%) | - | - | - | - | - |
|  | Hypertension - no. (%) | | 5380 (59.9%) | 1380 (61.6%) | 1141 (59.1%) | 1201 (57.8%) | 1564 (60.5%) | 3403 (37.9%) | 892 (41.9%) | 874 (34.7%) | 840 (35.3%) | 758 (40.4%) |
|  | Blood pressure - mm Hg | |  |  |  |  |  |  |  |  |  |  |
|  |  | Systolic | 148±21.3 | 149±21.9 | 147±20.9 | 148±20.6 | 149±21.3 | 133±18.5 | 133±19.3 | 133±18.4 | 133±17.8 | 134±18.7 |
|  |  | Diastolic | 86.5±12.4 | 87.0±13.2 | 85.3±12.1 | 86.6±11.8 | 86.9±12.0 | 80.0±10.7 | 79.2±11.3 | 79.8±10.6 | 80.2±10.3 | 81.1±10.6 |
|  | Cholesterol - mmol/litre | |  |  |  |  |  |  |  |  |  |  |
|  |  | HDL | 1.15±0.35 | 1.18±0.37 | 1.15±0.37 | 1.14±0.34 | 1.12±0.33 | 1.22±0.37 | 1.22±0.38 | 1.23±0.38 | 1.21±0.35 | 1.20±0.37 |
|  |  | LDL | 2.97±1.01 | 3.03±1.08 | 2.95±0.98 | 2.98±0.98 | 2.91±0.98 | 2.97±0.97 | 2.97±1.01 | 3.04±0.97 | 2.97±0.94 | 2.90±0.92 |
|  | Diabetes mellitus - no. (%) | | 1529 (17.0%) | 359 (16.0%) | 320 (16.6%) | 361 (17.4%) | 461 (17.8%) | 1142 (12.7%) | 291 (13.7%) | 311 (12.3%) | 305 (12.8%) | 223 (11.9%) |
|  | AFIB/Atrial Flutter - no. (%) | | 972 (10.8%) | 306 (13.7%) | 212 (11.0%) | 172 (8.27%) | 255 (9.86%) | 281 (3.13%) | 78 (3.66%) | 72 (2.86%) | 61 (2.56%) | 66 (3.51%) |
|  | Diuretic Pre-admission - no. (%) | | 1168 (13.0%) | 300 (13.4%) | 262 (13.6%) | 233 (11.2%) | 348 (13.5%) | 810 (9.01%) | 200 (9.40%) | 188 (7.47%) | 209 (8.79%) | 202 (10.8%) |
|  | Diuretic In Hospital - no. (%) | | 2070 (23.0%) | 540 (24.1%) | 421 (21.8%) | 446 (21.5%) | 603 (23.3%) | 366 (12.9%) | 78 (11.7%) | 82 (10.3%) | 97 (13.8%) | 106 (16.5%) |
|  | Current smoker - no. (%) | | 2674 (29.8%) | 619 (27.6%) | 595 (30.8%) | 668 (32.1%) | 761 (29.4%) | 1878 (20.9%) | 439 (20.7%) | 525 (20.8%) | 510 (21.4%) | 392 (20.9%) |

## Supplementary Table 5 - Association of estimated 24-hour sodium excretion and risk of ***ischaemic*** stroke

| **Estimated Sodium Excretion** | | | | | | |
| --- | --- | --- | --- | --- | --- | --- |
|  | | | <2.8 g/day | 2.8-3.5 g/day | 3.5-4.2 g/day | >4.2 g/day |
|  | | | (N=3618) | (N=3718) | (N=3790) | (N=3599) |
| Analysis – odds ratio (95% CI) | | | | | | |
|  | Univariate analysis * | | 1.37 (1.24-1.51) | 1.00 | 1.08 (0.98-1.19) | 1.68 (1.52-1.85) |
|  | Multivariate analysis | | | | | |
|  |  | Analysis including age and BMI | 1.37 (1.24-1.52) | 1.00 | 1.08 (0.98-1.19) | 1.70 (1.53-1.88) |
|  |  | Primary analysis † | 1.36 (1.22-1.52) | 1.00 | 1.07 (0.96-1.19) | 1.67 (1.50-1.87) |
|  |  | Analysis including dietary score and potassium ‡ | 1.16 (1.03-1.30) | 1.00 | 1.26 (1.12-1.40) | 2.53 (2.24-2.86) |
|  |  | Analysis including HTN and medications which modify sodium excretion § | 1.12 (0.99-1.28) | 1.00 | 1.23 (1.08-1.39) | 2.44 (2.13-2.79) |

## Supplementary Table 6 - Association of estimated 24-hour sodium excretion and risk of ***intracerebral haemorrhage***

| **Estimated Sodium Excretion** | | | | | | |
| --- | --- | --- | --- | --- | --- | --- |
|  | | | <2.8 g/day | 2.8-3.5 g/day | 3.5-4.2 g/day | >4.2 g/day |
|  | | | (N=1086) | (N=989) | (N=918) | (N=1123) |
| Analysis – odds ratio (95% CI) | | | | | | |
|  | Univariate analysis * | | 1.59 (1.32-1.92) | 1.00 | 1.45 (1.20-1.76) | 2.55 (2.11-3.08) |
|  | Multivariate analysis | | | | | |
|  |  | Analysis including age and BMI | 1.63 (1.34-1.98) | 1.00 | 1.43 (1.18-1.74) | 2.54 (2.09-3.08) |
|  |  | Primary analysis † | 1.62 (1.32-1.99) | 1.00 | 1.39 (1.13-1.72) | 2.38 (1.93-2.92) |
|  |  | Analysis including dietary score and potassium ‡ | 1.61 (1.31-1.99) | 1.00 | 1.39 (1.12-1.72) | 2.28 (1.82-2.84) |
|  |  | Analysis including HTN and medications which modify sodium excretion § | 1.33 (0.99-1.78) | 1.00 | 1.30 (0.96-1.74) | 1.88 (1.38-2.57) |

## Supplementary Table 7 – Multivariate (Conditional) Association Between Estimated 24-Hour Urinary Sodium Excretion and Stroke in Subgroups

|  | <2.8 g/day | 2.8-3.5 g/day | 3.5-4.26 g/day | >4.26 g/day | P |
| --- | --- | --- | --- | --- | --- |
|  | N=4751 | N=4750 | N=4750 | N=4750 |  |
| European (5885) | 1.57 (1.30-1.89) | 1.00 | 0.85 (0.71-1.03) | 1.34 (1.11-1.63) | <0.001 |
| Chinese (8009) | 1.16 (0.98-1.36) | 1.00 | 1.28 (1.10-1.47) | 1.85 (1.59-2.14) |  |
| Other Asian (1397) | 1.06 (0.70-1.62) | 1.00 | 1.91 (1.22-2.97) | 10.83 (6.35-18.48) |  |
| Arab (502) | 2.51 (1.13-5.56) | 1.00 | 1.55 (0.57-4.18) | 2.18 (0.71-6.72) |  |
| Latin American (2812) | 1.40 (1.06-1.87) | 1.00 | 0.90 (0.67-1.20) | 1.61 (1.23-2.13) |  |
| Black African (993) | 2.21 (1.37-3.55) | 1.00 | 1.31 (0.79-2.19) | 1.95 (1.10-3.47) |  |
| Other (1114) | 2.21 (1.43-3.41) | 1.00 | 0.90 (0.54-1.50) | 1.96 (1.12-3.43) |  |
|  |  |  |  |  |  |
| BMI >30 (3514) | 1.14 (0.68-1.93) | 1.00 | 0.95 (0.58-1.55) | 1.35 (0.81-2.26) | 0.009 |
| BMI <=30 (17522) | 1.33 (1.19-1.48) | 1.00 | 1.15 (1.03-1.28) | 1.92 (1.72-2.15) |  |
|  |  |  |  |  |  |
| Male (12353) | 1.43 (1.26-1.62) | 1.00 | 1.14 (1.01-1.29) | 1.83 (1.62-2.07) | 0.8845 |
| Female (8812) | 1.34 (1.15-1.56) | 1.00 | 1.11 (0.95-1.29) | 1.79 (1.54-2.09) |  |
|  |  |  |  |  |  |
| Age >75 (3929) | 1.32 (1.04-1.68) | 1.00 | 1.17 (0.90-1.51) | 1.78 (1.38-2.30) | 0.41 |
| Age <=75 (17236) | 1.41 (1.27-1.58) | 1.00 | 1.12 (1.01-1.24) | 1.84 (1.65-2.05) |  |
|  |  |  |  |  |  |
| Previous HTN (10485) | 1.19 (0.99-1.44) | 1.00 | 1.08 (0.89-1.31) | 1.51 (1.25-1.82) | 0.22 |
| No HTN (10679) | 1.43 (1.19-1.73) | 1.00 | 1.25 (1.04-1.49) | 1.92 (1.59-2.32) |  |
|  |  |  |  |  |  |
| Diuretic (2387) | 1.55 (0.71-3.41) | 1.00 | 1.06 (0.53-2.12) | 1.46 (0.77-2.76) | 0.1505 |
| No Diuretic (18766) | 1.40 (1.26-1.56) | 1.00 | 1.14 (1.03-1.27) | 1.87 (1.68-2.09) |  |

The primary model included age, BMI, education level, alcohol, diabetes at baseline, atrial fibrillation/flutter at baseline, smoking and physical activity level.

## Supplementary Figure 1 – Association of Estimated 24-hr sodium excretion with risk of all stroke (Below Median Potassium)


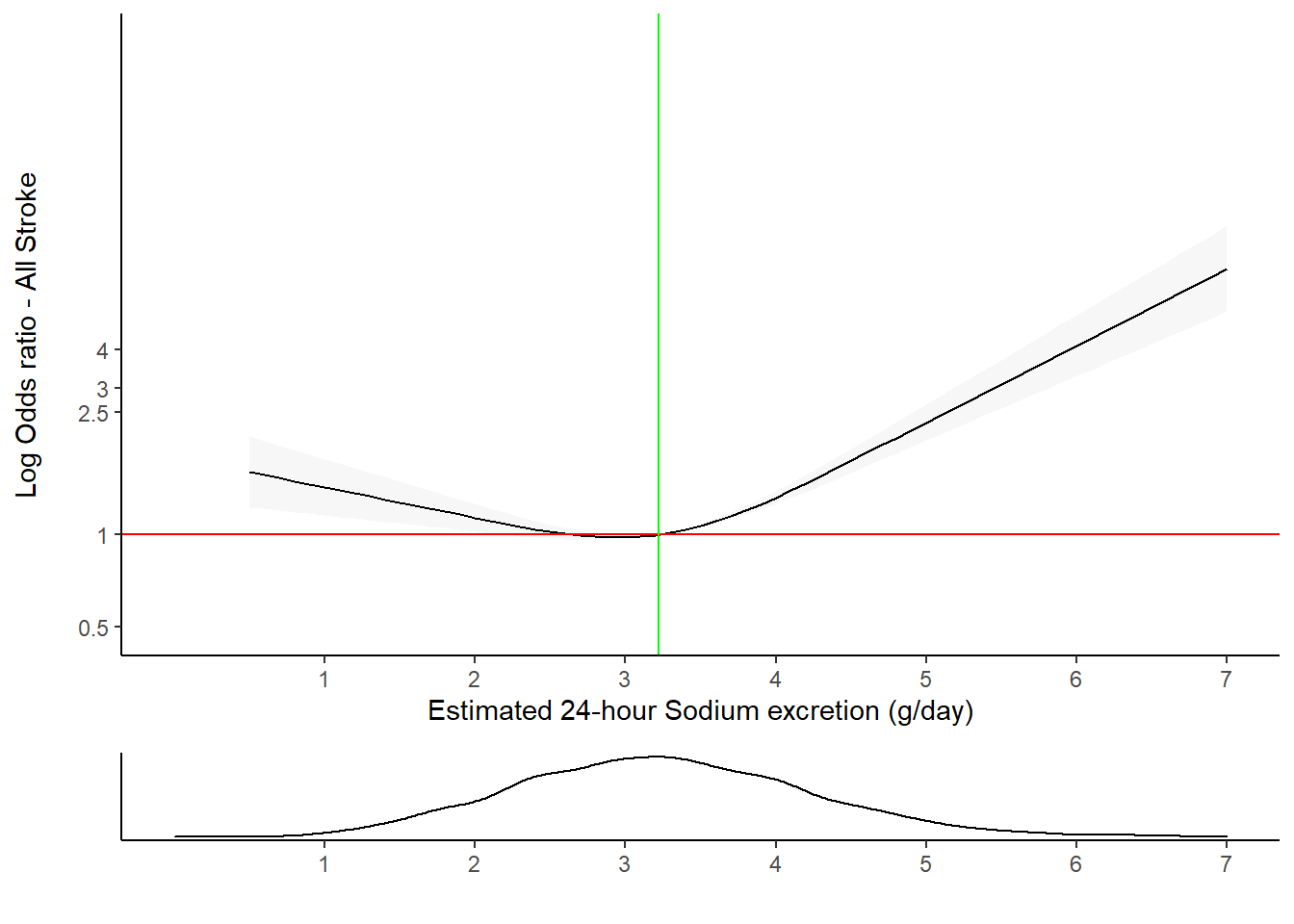


The primary model included age, BMI, education level, alcohol, diabetes at baseline, atrial fibrillation/flutter at baseline, smoking and physical activity level.

## Supplementary Figure 2 – Association of Estimated 24-hr sodium excretion with risk of all stroke (Above Median Potassium)

**
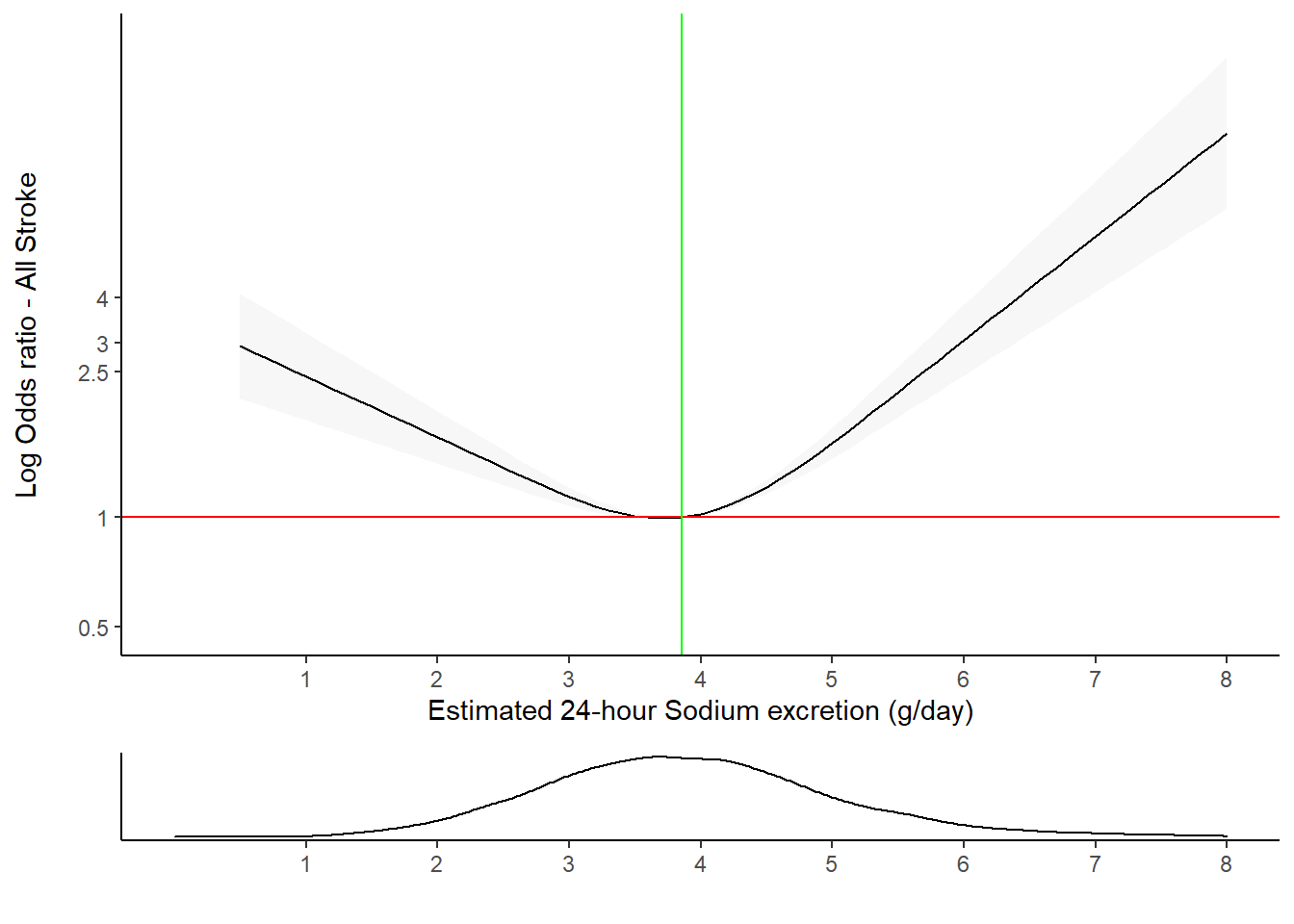
**

The primary model included age, BMI, education level, alcohol, diabetes at baseline, atrial fibrillation/flutter at baseline, smoking and physical activity level.

## Supplementary Figure 3 – Association of Estimated 24-hr sodium excretion (Kawasaki) with risk of all stroke


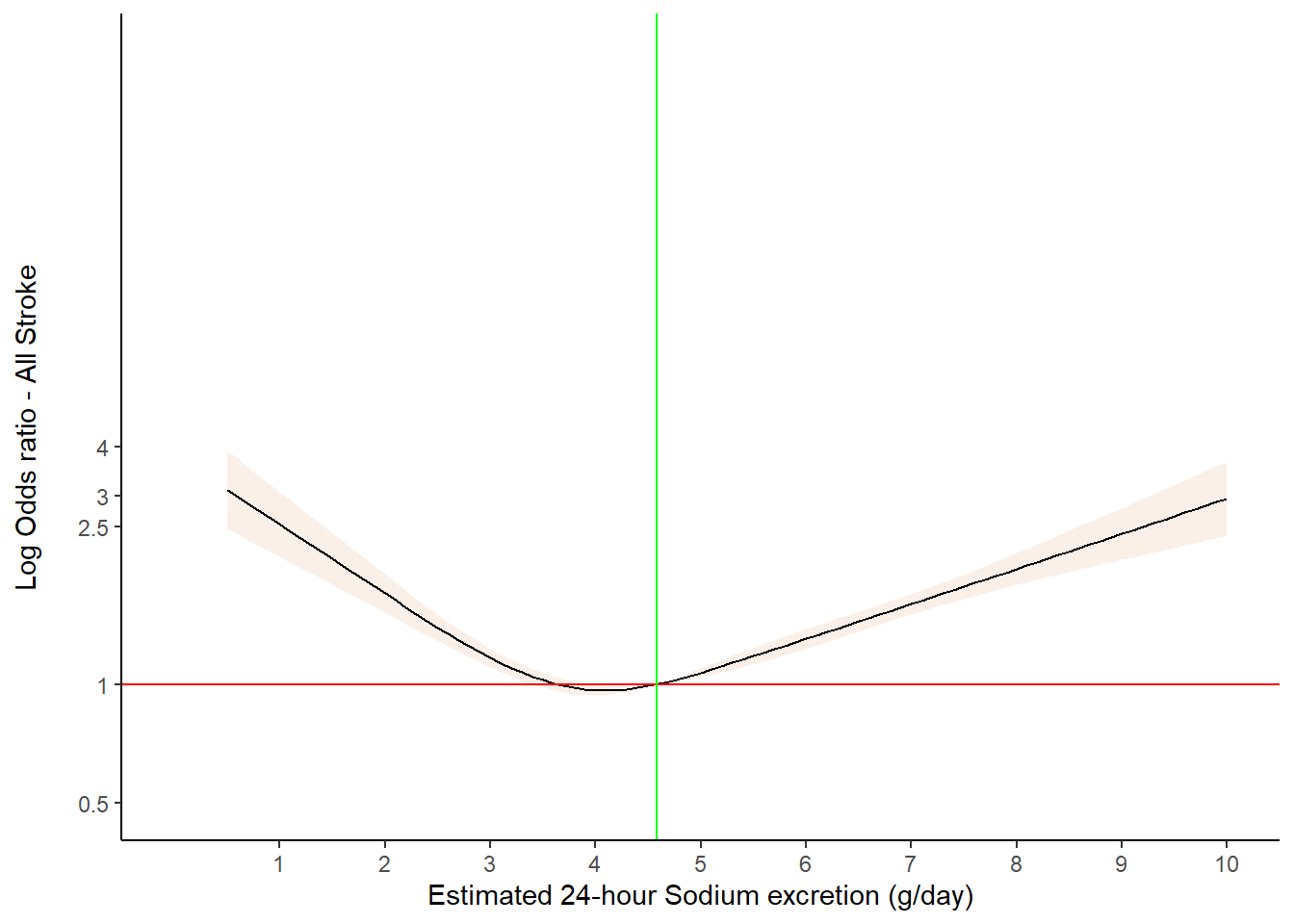


## Supplementary Figure 4 – Association of Estimated 24-hour sodium excretion (Kawasaki) with risk of Ischaemic stroke


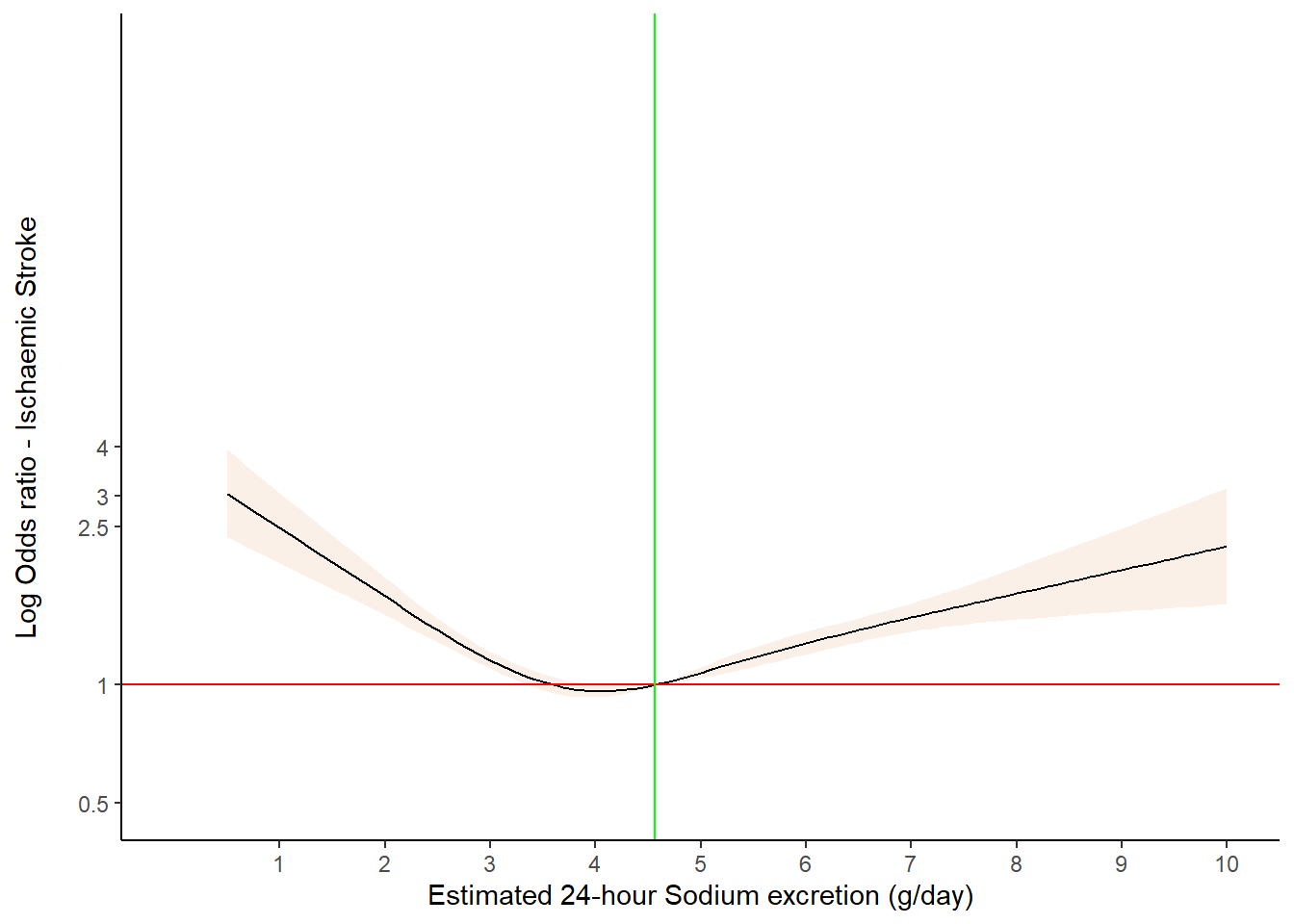


## Supplementary Figure 5 – Association of Estimated 24-hour sodium excretion (Kawasaki) with risk of ICH


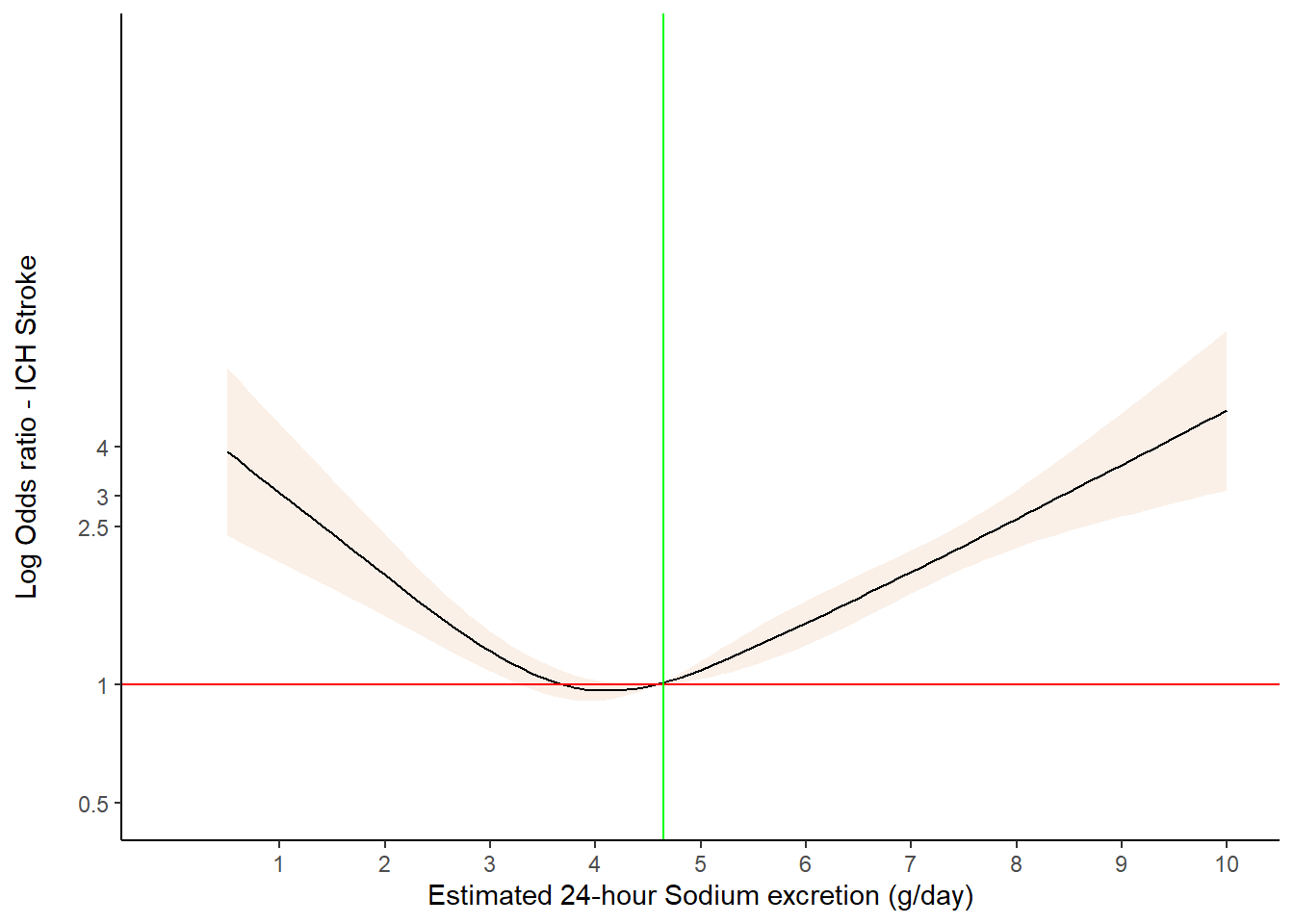

Supplement: hpaa176_suppl_Supplementary_Appendix [file hpaa176_suppl_supplementary_appendix.docx]
